# Quantifying the Time-Lag Effects of Human Mobility on the COVID-19 Transmission: A Multi-City Study in China

**DOI:** 10.1109/ACCESS.2020.3038995

**Published:** 2020-11-18

**Authors:** Wang Xi, Tao Pei, Qiyong Liu, Ci Song, Yaxi Liu, Xiao Chen, Jia Ma, Zhixin Zhang

**Affiliations:** 1 State Key Laboratory of Resources and Environmental Information System, Institute of Geographical Sciences and Natural Resources ResearchChinese Academy of Sciences 12381 Beijing 100101 China; 2 College of Resources and EnvironmentUniversity of Chinese Academy of Sciences Beijing 100049 China; 3 Jiangsu Center for Collaborative Innovation in Geographical Information Resource Development and Application Nanjing 210023 China; 4 State Key Laboratory of Infectious Disease Prevention and Control, Collaborative Innovation Center for Diagnosis and Treatment of Infectious Disease, National Institute for Communicable Disease Control and PreventionChinese Center for Disease Control and Prevention 12415 Beijing 102206 China; 5 Dongfang HospitalBeijing University of Chinese Medicine 47839 Beijing 100078 China; 6 China-Japan Friendship Hospital 36635 Beijing 100029 China

**Keywords:** COVID-19, control measures, cross correlation, human mobility, influencing factors

## Abstract

The first wave of the 2019 novel coronavirus (COVID-19) epidemic in China showed there was a lag between the reduction in human mobility and the decline in COVID-19 transmission and this lag was different in cities. A prolonged lag would cause public panic and reflect the inefficiency of control measures. This study aims to quantify this time-lag effect and reveal its influencing socio-demographic and environmental factors, which is helpful to policymaking in controlling COVID-19 and other potential infectious diseases in the future. We combined city-level mobility index and new case time series for 80 most affected cities in China from Jan 17 to Feb 29, 2020. Cross correlation analysis and spatial autoregressive model were used to estimate the lag length and determine influencing factors behind it, respectively. The results show that mobility is strongly correlated with COVID-19 transmission in most cities with lags of 10 days (interquartile range 8 – 11 days) and correlation coefficients of 0.68 ± 0.12. This time-lag is consistent with the incubation period plus time for reporting. Cities with a shorter lag appear to have a shorter epidemic duration. This lag is shorter in cities with larger volume of population flow from Wuhan, higher designated hospitals density and urban road density while economically advantaged cities tend to have longer time lags. These findings suggest that cities with compact urban structure should strictly adhere to human mobility restrictions, while economically prosperous cities should also strengthen other non-pharmaceutical interventions to control the spread of the virus.

## I. Introduction

The novel coronavirus disease 2019 (COVID-19), first identified in Wuhan, Hubei province, in December 2019, has spread rapidly across China and even globally [1], [2]. On March 11, The World Health Organization (WHO) declared the COVID-19 outbreak a pandemic, which has posed a threat to global public health. 43,623,111 cases of COVID-19, including 1,161,311 deaths, have been reported globally as of October 27, 2020 [3]. In the absence of effective vaccine and specific therapeutic drug, many countries have implemented non-pharmaceutical interventions (NPIs), of which human mobility restrictions is an essential component [4]–[6].

Several studies have investigated the effect of human mobility and control measures on the COVID-19 pandemic around the world [7]–[9]. Human mobility is one of the key factor in the spread of infectious diseases [10], [11]. Transmission of the disease is enabled by both the long-distance transportation, such as air, land and sea transportation among countries [12], and the short trip such as commuting within cities [13]. Based on a “risk source” model, Jia *et al.* [14] found that the population outflow from Wuhan could accurately predict the relative frequency and geographic distribution of COVID-19 incidence through February 19, 2020 in China. To contain the spread of COVID-19, mobility restrictions are implemented in many regions. Chinazzi *et al.* [15] estimate the travel ban from Wuhan on January 23 led to a 77% reduction in cases imported from China to the rest of the world in early February; Gatto *et al.* [16] found that restrictions posed to mobility have reduced the COVID-19 transmission by 45% in Italy.

These studies demonstrate that human mobility restrictions can effectively mitigate the spread of COVID-19. However, the effect of such restrictions on the COVID-19 transmission is not immediately evident. For example, Badr *et al.* [17] found that the effect of decreased mobility on COVID-19 transmission in the US took at least 9-12 days and even 3 weeks to be perceptible, consistent with the incubation period plus additional time for reporting. Therefore the length of this time-lag indicates the efficacy of the measures. Quantifying this time-lag is important for relieving public panic (for example, people care about how soon the COVID-19 transmission will decline after mobility has declined) and uncovering the factors influencing it can, in turn, inform policy development and refinement. However, until recently, little is known about these. To address these issues, in this study, we aimed to investigate the effect of reduced human mobility on COVID-19 transmission in severely affected Chinese cities during the first wave of COVID-19 epidemic in China, based on the assumption that the decline in new case solely depends on the mobility. The cross-correlation analysis were used to quantify the time-lag, socio-demographic and environmental factors influencing the time-lagged effects were explored using both multiple linear regression model and spatial autoregressive model.

The remainder of this paper is organized as follows. Section II introduces the study area and data. Section III shows our analytical framework. Section IV shows the results of our analysis, and a discussion is provided in section V. The conclusions are drawn in the last section.

## II. Study Area and Data

We chose China as the study area. Despite being the first place to be hit by COVID-19, China has managed to control this epidemic rapidly and effectively. In order to prevent the spread of the epidemic, Wuhan was placed under a strict lockdown from Jan 23. All provinces launched the Level 1 Response almost simultaneously, with the benefit of a centralized epidemic response system. The government implemented control measures such as the isolation of suspected and confirmed cases, suspension of the public transportation system and so on. People were encouraged to stay at home and keep social distancing. Therefore, human movements were severely curtailed.

**FIGURE 1.**
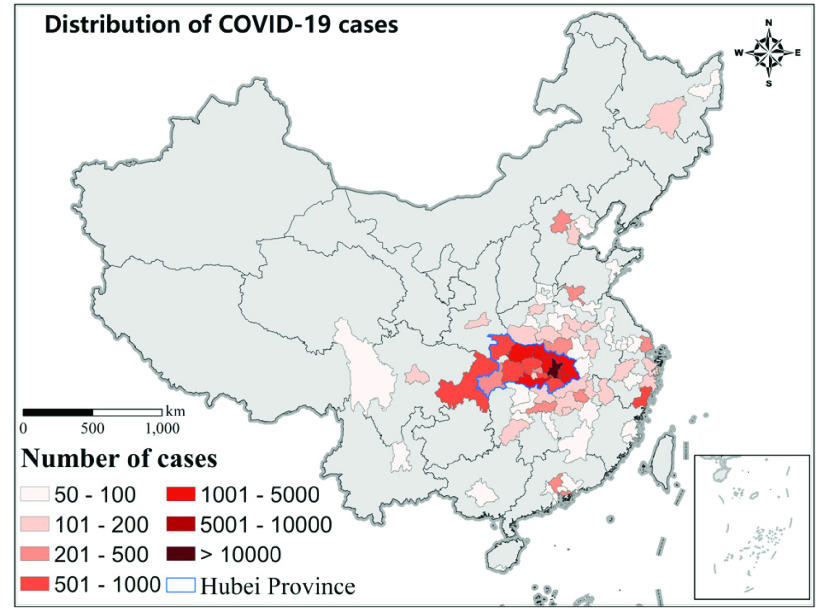
Cities with more than 50 cases of COVID-19 from Jan 17 to Feb 29, 2020.

We collected the daily count of new confirmed cases from the official reports of the health commissions in corresponding provinces or cities from January 17 (one week before the Wuhan lockdown) to February 29, 2020. After February 29, the international importation case become predominant in China [18], so we chose the time period before that. We recorded the date when the Level 1 Response was launched for each province. This key date marked the beginning of the mobility restrictions. Besides, we also recorded each city’s epidemic duration, defined as the interval between the date of first case occurrence and the first date that new confirmed cases remain zeros in 7 consecutive days. We choose city with more than 50 confirmed cases as our study area (80 cities in total, 62 cities excluding Hubei province). 50 is the upper quartile of all cities’ cases, therefore we choose this as a threshold to represent severely affected cities during the first wave of COVID-19 epidemic in China, as illustrated in Fig. 1.

The Baidu mobility index data during the same study period was acquired from Baidu Map (https://qianxi.baidu.com/). Baidu records users’ location based on the GPS, IP address and WI-FI when they use Baidu apps on their phones, such as mapping and searching. As of 2017, Baidu mapping service had a 30% market share in China [19]. We used the mobility index of intra-city traffic volume to represent the human mobility. The Baidu mobility index for city $i$ on date $d$ is calculated as:

\begin{equation*} \mathrm {M}_{i,d}=\frac {a_{i,d}}{b_{i}}\times C\tag{1}\end{equation*}

where $a_{i,d}$ is the number of people travelling within city $i$ on date $d$. It also implicitly includes people who enter city $i$ and generate an origin and destination pair in city *i.* Hence both short- and long-distance mobility are considered in $a_{i,d}$. $b_{i}$ is the number of residents in the city *i,* $C$ is a constant and is same for all cities. Therefore, the Baidu mobility index is a ratio considering the size of city and can be compared across cities. In this dataset, the maximum value of mobility index appeared in Bozhou city (a small city in Anhui province) on January 23, at 8.8778, and the minimum value appeared in Wuhan city (a core city in Hubei province) on February 22, at 0.5687. Recent studies have shown that this index can be used to represent residents’ mobility and the resumption of economic activities [20]–[22].

**FIGURE 2.**
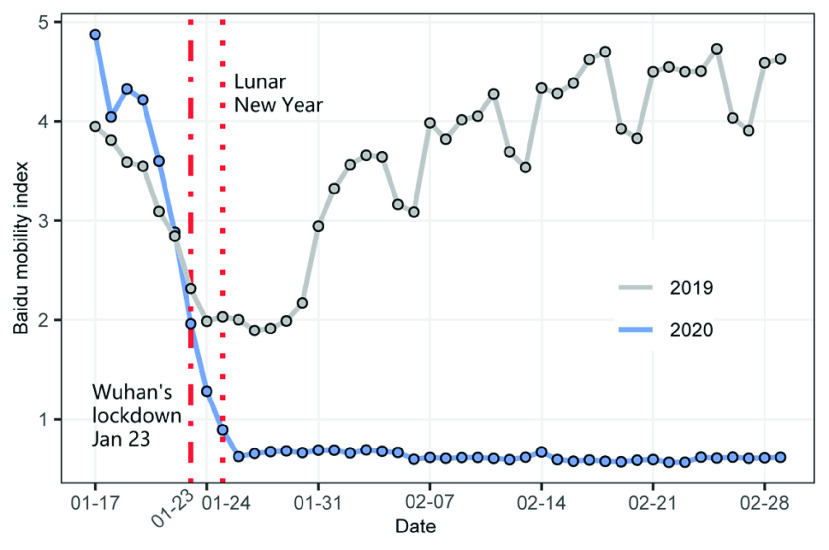
Baidu mobility index for Wuhan.

Figure 2 shows the Baidu mobility index for Wuhan from January 17 to February 29, 2020 and for the same period in the 2019 lunar calendar. The study period includes the Spring Festival, which is a traditional holiday for family reunions and public gatherings. Due to social distancing, people turn to stay at home during this holiday in 2020, hence the substantially decrease in the mobility index compared to 2019.

To understand the city-level variation in the time lag between human mobility and COVID-19 transmission, we collected socio-demographic and environmental data for cities. Population density, family size, the proportion of elderly people (aged 60 and above) and GDP per capita data for each city were retrieved from the China City (Prefecture) Statistical Yearbook 2018 (produced by the National Bureau of Statistics of China). To calculate road density in the urban area of each city, we collected the road network data in 2018 from one of the biggest navigation companies in China. The urban boundary data in 2018 were acquired from [23], which were generated using 30 m global artificial impervious area data [24]. We also collected the density of designated hospitals for diagnosing COVID-19 infection in each city. To measure the impact of the population outflow from Wuhan, we collected mobile-phone-data-based volume of outbound traffic from Wuhan to other cities from January 1 to January 23, 2020, for details about this data please refer to [14]. Table 1 summarizes the descriptive statistics for all data used in this study.

**TABLE 1:** Descriptive Statistics for Data Used in this Study

Variable	Mean(SD)
Number of cases(count)	246.8(2743.9)
Baidu mobility index	3.4(1.5)
Population density(persons/km^2^)	660.7(406.4)
Family size(persons)	3.2(0.4)
GDP per capita(yuan)	86038.6(42329.4)
Urban road density(km/km^2^)	9.0(7.4)
Number of designated hospitals(count)	8.8(7.2)
Total movement from Wuhan(count)	32267.6(40352.3)
Timing of Level 1 Response(day)	1.0(0.8)
Area of city(km^2^)	15989.0(21179.0)
Proportion of elderly people(%)	17.1(4.8)

## III. Analytical Framework

**FIGURE 3.**
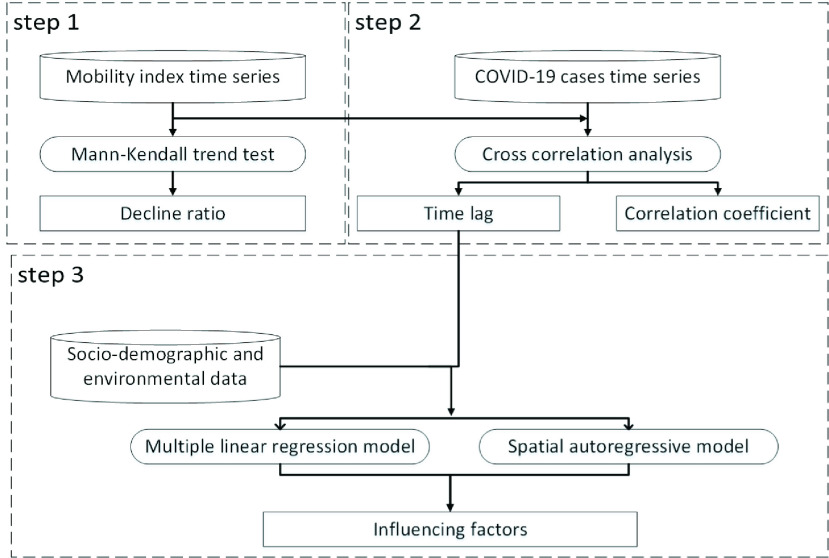
The flowchart of the analytical framework.

Figure 3 shows the analytical framework in this study. It is composed of three steps. In the first step, we analyzed the mobility time series to evaluate the impact of the control measures on residents’ mobility. Second, we performed a cross correlation analysis between the human mobility and the COVID-19 new case time series to explore the time lag and correlation coefficient between them. Third, we used both multiple linear regression model and spatial autoregressive model to explore the potential factors affecting this relationship. The following section will present the procedure of analyzing mobility time series and cross correlation, taking a typical city (Ningbo with 157 confirmed cases until February 29) in China as an example. Then the selection of variables and construction of the multiple linear regression model will be introduced.

### A. Analysis of Mobility Time Series

To evaluate the impact of the control measures on human mobility, we first found the date when Level 1 Response was launched in the city and further extracted the mobility time series one week before and after the date for analysis. We used the Mann-Kendall trend test [25] to detect the monotonic trend of mobility time series in the first 14 days. The decline ratio (DR) was defined as:

\begin{equation*} \mathrm {DR}=100 \times \frac {M_{b}-M_{a}}{M_{b}}\tag{2}\end{equation*}

where M_*b*_ and M_*a*_ are the median of two subseries before and after the date of Level 1 Response. This value quantified the magnitude of mobility change in response to the control measures; a large value of DR showed a great reduction.

**FIGURE 4.**
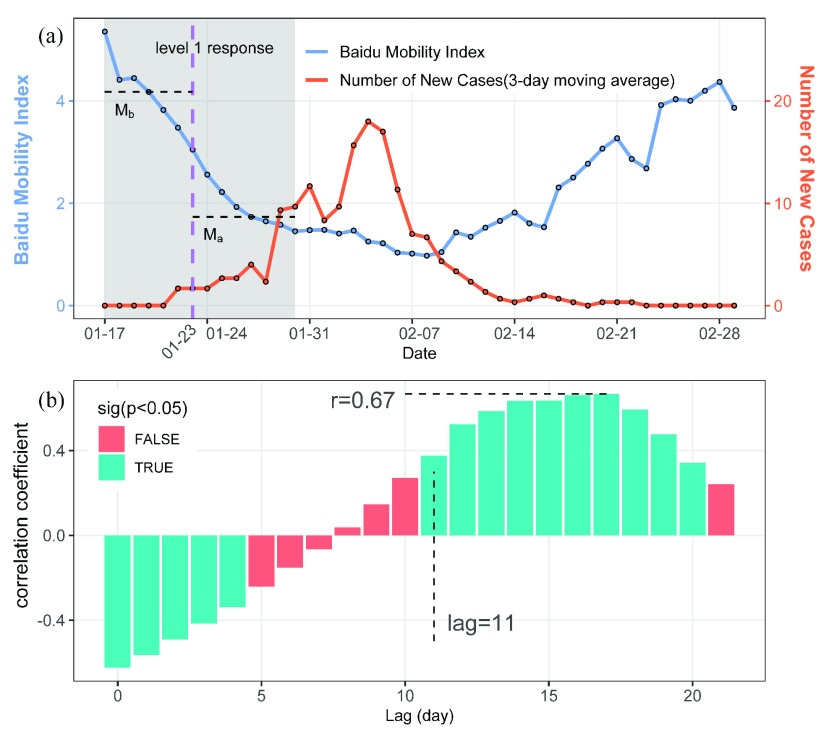
The analytical framework for Ningbo city. (a) Time series for Baidu mobility index and COVID-19 case. The dashed purple line shows the date when Level 1 Response was launched. (b) Time-lag correlation between mobility and COVID-19 cases series.

Taking Ningbo as an example, as illustrated in Fig. 4(a), human mobility showed a declining trend in the first 14 days(S = −89, p < 0.0001) with a decline ratio of 58% ($\mathrm {M}_{b}= 4.18$, $\mathrm {M}_{a}= 1.73$) when the Level 1 Response was launched in Ningbo. We performed this analysis for all cities with more than 50 cases and a correlation analysis between DR and the total movements from Wuhan before lockdown in each city. The results will be presented in section IV-A.

### B. Cross Correlation Between Human Mobility and COVID-19 Cases

To quantify the relationship between human mobility and COVID-19 transmission, we performed a cross correlation analysis between the two time series. Since the daily case reporting data might have errors due to both reporting issues and limited test capacity, we firstly used 3-day moving averages to smooth them [17]. Then we calculated cross-correlation coefficient ($\mathrm {r}_{k}^{xy}$) between the mobility time series and the corresponding COVID-19 case time series with lag from 0 to 21 days. $\mathrm {r}_{k}^{xy}$ is calculated as:

\begin{equation*} \mathrm {r}_{k}^{xy}=\frac {\sum {(y_{t}-\bar {y})(x_{t-k}-\bar {x})}}{\sqrt { {\sum {(y_{t}-\bar {y})}^{2} }}\sqrt {\sum {(x_{t}-\bar {x})}^{2}}}\tag{3}\end{equation*}

where $x_{t}$ is the mobility time series, $y_{t}$ is the COVID-19 daily new case time series, $\bar {x}, \bar {y}$ are the mean values of each series, and k is the lag [26]. The cross correlation method have been widely used in estimating the lag between two time series in previous studies [27], [28]. The first positive significant time-lag (denote by K) reflects the time interval between the decline in COVID-19 transmission in response to the mobility decline in the city. The maximum correlation coefficient denotes how well the mobility series predicts the new case series. Therefore, we use both the first positive significant time-lag and the maximum correlation coefficient to quantify the relationship between human mobility and COVID-19 transmission. We further performed a correlation analysis between the first positive significant time-lag and epidemic duration to see whether cities with faster response will have a shorter epidemic duration.

As shown in Fig. 4(b), the relationship between two time series in Ningbo changed from a significant negative correlation to a positive correlation as the lag increases, which indicates that the effect of decline in human mobility on COVID-19 transmission is not immediately evident. When the lag was 11 days, they began to show a significant positive correlation (r > 0.3, p < 0.05). The maximum correlation coefficient was 0.67, which suggested that the reduction of COVID-19 new case were associated well with the decline of human mobility. The time series of mobility and new cases for all 80 cities are shown in the supplementary material. We performed the cross correlation analysis for all 80 cities, the results of the lag and correlation coefficient will be presented in section IV-B.

### C. Multiple Linear Regression Model

To explore the factors associated with the time lag between human mobility and COVID-19 transmission, a MLR model was developed as:

\begin{equation*} \mathrm {K}_{i}=\beta _{0}+x_{i}\beta +\varepsilon _{i}\tag{4}\end{equation*}

where in the city $i$, K_*i*_ is the first positive significant time-lag, as introduced in section III-B, $x_{i}$ is the vector of explanatory variables, $\beta $ is the vector of regression coefficients, and $\varepsilon _{i}$ is a random error term. The potential explanatory variables are listed in Table 2. The reasons for choosing these variables are as follows. First, recent studies show that elderlies are found to be more vulnerable to COVID-19 [29], therefore we use the proportion of elderly people (aged 60 and over) to reflect the age structure of the local population. Second, many COVID-19 transmission were found to be within family clusters in China [30], [31], we wondered if the larger family size would contribute to the spread of COVID-19 so that delay the response time. Third, road density in urban area reflected the city’s urban structure [32], which has impact on the population flow within the city and the COVID-19 spread [33]. Fourth, the timing of Level 1 response may also be a key factor in mitigating the spread, we wondered whether the earlier the policy were launched, the faster response between mobility decline and case decline. To compare the degree of influence of each impact factor on the dependent variable, all variables were normalized before the regression. The MLR results for time lag will be presented in section IV-C.

**TABLE 2:** Information About the Potential Factors

Type	Factor	Description
Socio-demographic variables	Population density (persons/km^2^)	Household registered population in the city/area of the city
	Family size (persons)	Average household size
	Proportion of elderly people(%)	The ratio of the older people(aged 60 and above) in the city
GDP per capita (yuan)	Gross regional domestic product by its total population
Environmental variables	Urban road density(km/km^2^)	The total length of roads in the urban area/urban area of the city
	Area of city(km^2^)	The area of administrative boundary of the city
	Density of designated hospitals	Density of designated hospitals for diagnosing the COVID-19 infection
Total movement from Wuhan	The volume of traffic from Wuhan to the city between January 1 and January 23 (mobile-phone-data-based)
Policy variables	Timing of Level 1 response	Number of days between the implementation of Level 1 response and January 23

### D. Spatial Autoregressive Model

The spatial dependence of time-lag are omitted from the MLR model, therefore we further applied a SAR(spatial autoregressive) model to account for this. The two most common models used in SAR are the SLM(spatial lag model) and SEM(spatial error model) [34]. In our case, we choose the spatial lag model based on the Lagrange multiplier principle [35]. The SLM uses a “spatial-lagged dependent variable” to incorporate spatial dependence into the regression model and was developed as:

\begin{equation*} \mathrm {K}_{i}=\beta _{0}+x_{i}\beta +\rho W_{i}\mathrm {K}_{i}+\varepsilon _{i}\tag{5}\end{equation*}

where $\rho $ is the spatial lag parameter and $W_{i}$ is a row of spatial weights matrix, which measures the spatial proximity of city $i$ to other cities. In this model, the weight matrix was generated based on the K-Nearest Neighbors to avoid the problem of isolates. The meaning of other variables is the same as equation (4). The SLM results for time lag will be presented in section IV-C.

All aforementioned data processing and statistical analyses were performed in Esri’s ArcGIS, GeoDa and the R (with packages of stats, car, gvlma, tseries) software environments. Results with p values of less than 0.05 were considered statistically significant in all statistical tests.

## IV. Results

### A. Change of Human Mobility in Response to Control Measures

**FIGURE 5.**
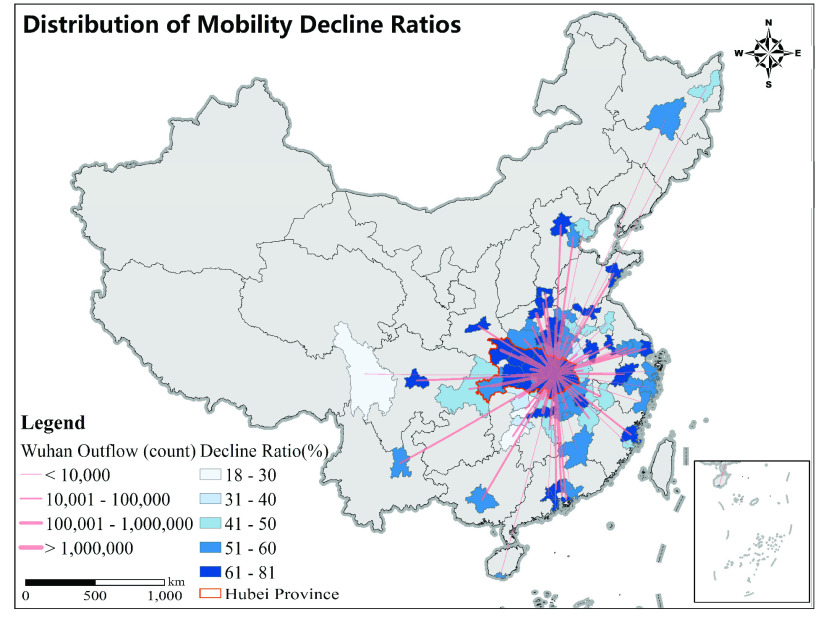
Mobility decline ratios for cities with more than 50 cases.

As shown in Fig. 5, all cities with more than 50 cases showed a statistically significant decreasing trend in mobility when the Level 1 Response was launched (p < 0.05). The decline ratio averaged 57.4% (SD = 11.3%, n = 80), with the maximum (81.3%) occurring in Wuhan. The decline ratio in each city was positively associated the total movements from Wuhan from January 1 to January 23 (Spearman’s rho = 0.52, p < 0.001, n = 79). At the early stage of the outbreak, the population outflow from Wuhan drives the distribution of COVID-19 cases. Therefore, this significant positive correlation suggests that in response to the outbreak, cities with a higher risk of imported COVID-19 infection had a more drastic decline in residents’ mobility.

### B. Distribution of Lag and Correlation Coefficient

**FIGURE 6.**
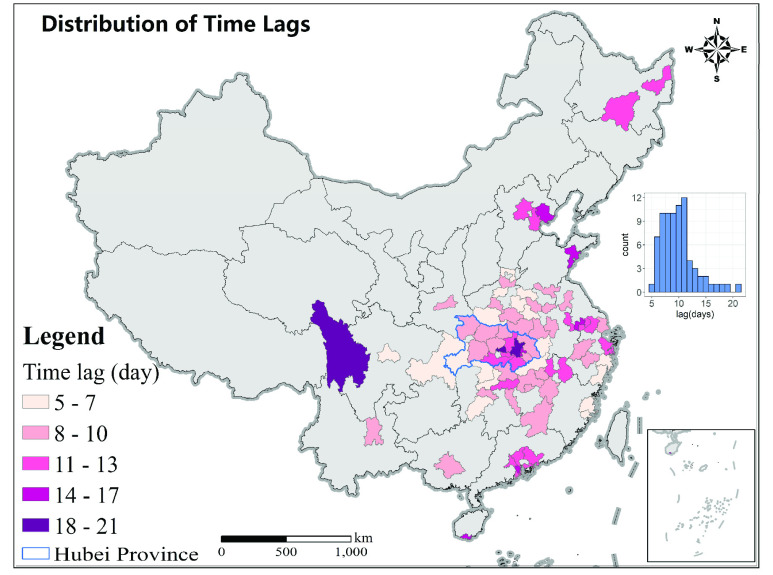
Time lags for cities with more than 50 cases. The inset shows the histogram for lag.

There was a significant positive lagged correlation (p < 0.05) between the mobility time series and COVID-19 new case in all cities except Jining, Qianjiang and Dongguan; therefore, we excluded them from the analysis of lag and correlation coefficient. As shown in Fig. 6, the median of time-lag is 10 days (interquartile range 8 – 11 days). This value is close to the total time from infection to confirmation on average, which is a combination of the incubation period (mean = 5.2 days [1], [36]) and the time from symptom onset to official reporting (median = 3 days [31]). A shorter lag means the decline in COVID-19 new case was more responsive to the decline in mobility. Cities in Hubei province had a longer lag (median = 10 days, interquartile range 8 – 11 days, n = 15) than those outside Hubei (median = 9 days, interquartile range 9 – 11 days, n = 62), which may be due to the limited testing and treatment resources at the early stage of the outbreak. The time-lag is positively related to the epidemic duration of cities (r = 0.4, p < 0.001, n = 77), which suggests that cities with faster response will also have a shorter epidemic duration.

**FIGURE 7.**
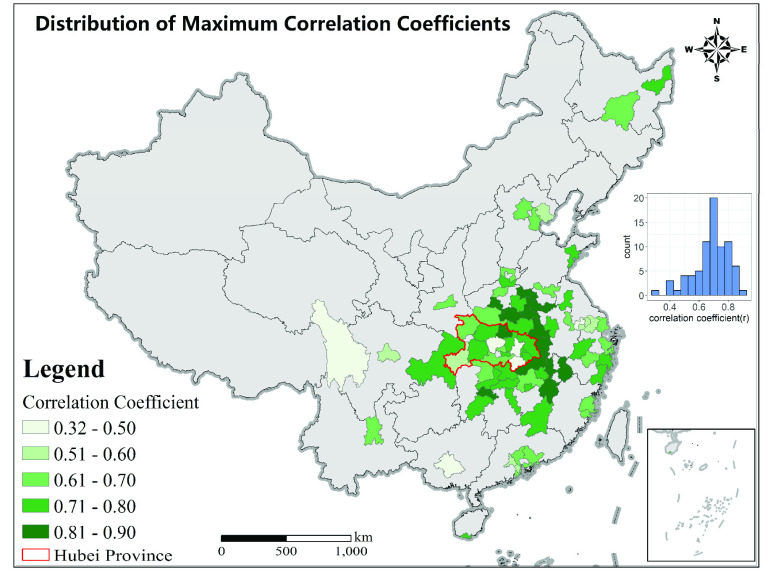
Correlation coefficients for cities with more than 50 cases. The inset shows the histogram for the correlation coefficient.

As shown in Fig. 7, cities that were close to the Hubei province generally had a larger correlation coefficient. The correlation coefficient averaged 0.69 (SD = 0.12, n = 77). The difference of correlation coefficient in and outside Hubei province is small (mean = 0.65 in Hubei and 0.70 outside Hubei). The coefficient of variations of the time-lag and correlation coefficients were 0.32 and 0.17, respectively. These indicated that although the time-lag varied across cities, the decline in mobility was consistently well associated with the decline in the COVID-19 cases.

### C. Factors Associated with Time Lag

The MLR and SLM results for time lag are reported in Table 3 and Table 4, respectively. We excluded cities in Hubei for their severe under-reporting bias. The regression models were both statistically significant (p < 0.001), and the SLM outperformed OLS based on the higher adjusted R^2^ (0.504) and a lower AIC (143.413). The results show that urban road density, density of designated hospitals and the volume of population flow from Wuhan had significant negative effects on the time lag, whereas GDP per capita had a significant positive effect. The significant positive sign of the autoregressive lag coefficient rho suggests that clustering of similar regions. The family size and proportion of elderly people might have a slight positive effect, but they are not statistically significant. The standardized coefficients of the significant influencing factors show that economy have greater influence on the time lag.

**TABLE 3:** The Results of Multiple Linear Regression for Lag

Variable	Standardized coefficient (beta)
Population density	0.019
Family size	0.155
GDP per capita	0.707^***^
Urban road density	−0.369^**^
Density of designated hospitals	−0.245^*^
Total movement from Wuhan(log)	−0.345^**^
Timing of Level 1 Response	0.017
Area of city	0.062
Proportion of elderly people	0.018
R^2^	0.555
Adjusted R^2^	0.478
AIC	144.718
F	7.211
P value	0.000
Sample size	62

^***^ p<0.001

^**^ p<0.01

^*^ p<0.05

**TABLE 4:** The Results of Spatial Lag Model for Lag

Variable	Standardized coefficient (beta)
Rho	0.285^*^
Population density	−0.032
Family size	0.156
GDP per capita	0.606^***^
Urban road density	−0.399^**^
Density of designated hospitals	−0.184^*^
Total movement from Wuhan(log)	−0.268^**^
Timing of Level 1 Response	−0.023
Area of city	0.071
Proportion of elderly people	0.017
R^2^	0.585
Adjusted R^2^	0.504
AIC	143.413
Sample size	62

^***^ p<0.001

^**^ p<0.01

^*^ p<0.05

## V. Discussion

### A. The Impact Mechanism of Time Lag

The time lag in this study reflects how long it takes for the effect of decline in mobility on COVID-19 transmission can be perceptible, and we found that a median of 10 days in Chinese cities with more than 50 cases. It can be regarded as a combination of the incubation period and the time from symptom onset to official reporting. The length of the time lag is influenced by a combination of factors, such as the transmission rate of virus, testing capacity, personal and government attitude towards the control measures. In this section we will discuss the joint effects of these factors on the time lag.

According to the results of MLR and SLM, we found that GDP per capita had a positive effect on time lag. This may be related to the fact that cities with higher per capita GDP generally have more social interactions as a result of more necessary economic activity [37], which may contribute to the transmission of COVID-19. With high transmission rates, an increasing number of infections will arise, new cases will continue to occur. It will delay the decline of new cases and therefore it leads to a long time lag. The COVID-19 outbreak posed a huge challenge to medical resources [38]. The demand for critical care, including hospital beds, intensive care units (ICU) and special medical facilities, is expected to increase with the rising number of cases. Cities with higher density of designated hospitals generally have better medical care quality and enough testing capacity, which enables the suspected and confirmed cases of COVID-19 to be treated immediately. This could help to reduce the epidemic duration so that has the negative effect on the time lag. At the early stage of outbreak, the population flow from Wuhan drives the distribution of COVID-19 cases [14]. Cities with close ties to Wuhan have a larger population influx and early detection of the imported cases. At the same time, as mobility declined, local transmission was contained and the epidemic curve peaked then declined rapidly, thus shortening the time lag. The road density in the urban area reflects the city organization, a higher road density usually means a more compact urban structure. Although the compact structure is believed to be more susceptible to the rapid spread of epidemics, but it also makes mobility restrictions quite effective, while the sprawled structure is less responsive to mobility restrictions [39]. This could help explain its negative effect on the lag in this study. The population density does not reflect the congestion level in the urban area, and both the differences in family size and the timing of Level 1 Response are small, therefore they are not significant.

In addition to these factors, there are some other important factors. However, they are difficult to quantify and therefore are not included in our regression models. Here, we briefly discuss the impact of these factors based on the results. First, the testing capacity is crucial at the early stage of the outbreak. As shown in Section IV-B, the testing capacity was limited in Hubei province at the very beginning of the outbreak and infected cases could not be diagnosed in time, therefore it has a longer time lag than regions outside of Hubei. At the same time, the attitude of local government was also an important factor. For example, Wenzhou was considered a city at higher infection risk due to its close ties with Wuhan, but the decisive decision-making and strict enforcement of control measures by the Wenzhou’s government led to a rapid decline in cases [40]. While in Jining city, the local government negligence made the Rencheng Prison a major source of infection and led to a spike in new case time series [41]. Therefore the peak of the epidemic curve was delayed. Although it is beyond the scope of this study to conduct detail analysis for these factors, they are critical to our understanding of time lag and need to be explored further in the future.

### B. Evaluation of Control Measures in Cities

**FIGURE 8.**
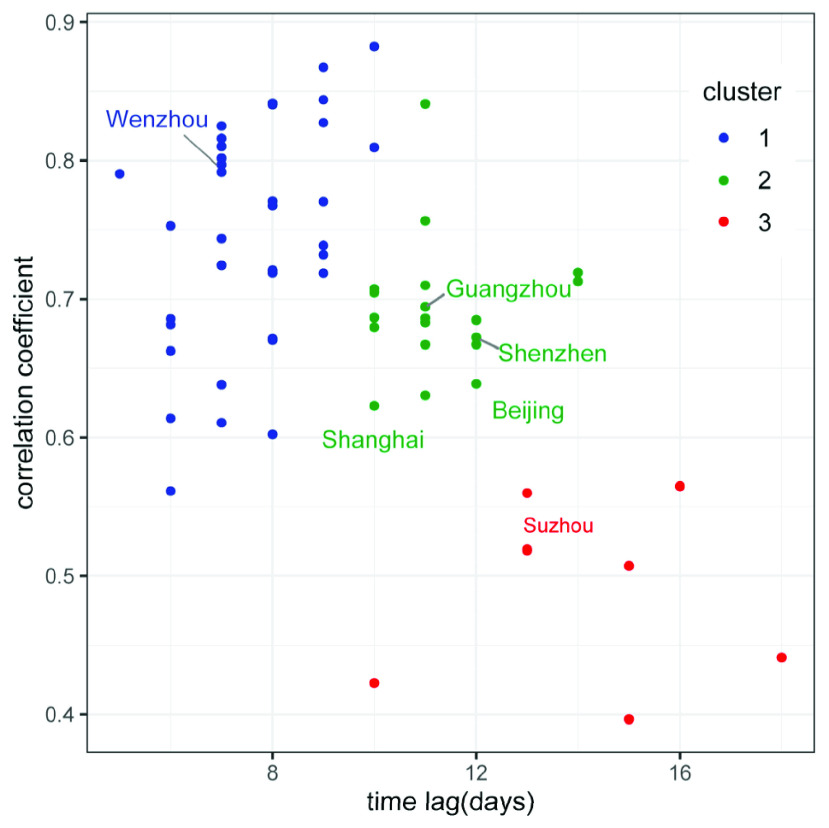
The cluster of cities based on lag and correlation coefficient.

The time lag and correlation coefficient could jointly be used to evaluate the effectiveness of mobility control measures implemented in cities, and also provide some implications for containing potential emerging infectious disease in the future. The scatterplot of lag and correlation coefficient is illustrated in Figure 8. After normalizing the two variables, we classified the cities into three clusters based on the K-means clustering.

Cities in cluster 1 have a short time lag and a high correlation coefficient, with the clustering center (lag = 7.6 days, r = 0.74). Mobility decline in these cities could predict the case decline well with a short lag, which indicated the mobility control measures have proven to be effective in containing COVID-19 outbreak. For example, because of its close economic ties with Wuhan, Wenzhou became the most affected Chinese city outside of Hubei at the early stage of the outbreak [40]. Control measures were preemptively implemented, such as the isolation of people who returned to Wenzhou from Wuhan, regional quarantine, and suspension of the public transportation system. These measures led to a large decline in mobility and the COVID-19 new case declined quickly in February. Its relative high road density in urban area and high medical care quality could contribute to the short lag. Cities in this cluster could focus on forestalling imported cases and gradually restoring the order of production and life.

Cities in cluster 2 have a medium time lag and a medium correlation coefficient, with the clustering center (lag = 7.6 days, r = 0.69). China’s megacities such as Beijing, Guangzhou, Shanghai and Shenzhen are all in this cluster. Take Beijing for example, it has a developed economy and a large floating population. Therefore Beijing has faced not only import risk from many other regions but also a high community transmission risk due to the frequent necessary economic activities. Therefore the emergency response should always be prepared to relaunch timely to reduce the risk of a secondary outbreak in this type of city.

Cities in cluster 3 have a long time lag and a low correlation coefficient, with the clustering center (lag = 14.1 days, r = 0.49). Mobility decline in these cities could not predict the case decline well and the lag is long. The longer lagged effect suggested that after the control measures were implemented, new infections still occurred for a relatively long time. The mobility decline ratios in these cities were the lowest of the three categories, the mobility restrictions played a limited role. The high proportion of elderly people and large city size may also contribute to the long time lag. Therefore some other non-pharmaceutical interventions should be enhanced, such as isolation of cases and close contacts.

Among the three clusters, more than half of the cities (69.4%) are concentrated in the cluster 1 and cluster 2. This relatively high proportion showed that the mobility control measures implemented in China effectively contained the COVID-19 outbreak. Decline in the mobility tend to be a strong signature for the decline in the COVID-19 transmission in the absence of a vaccine or effective antiviral.

## VI. Conclusion

In this study, we used cross-correlation analysis to quantify the time-lagged effect between the human mobility and the COVID-19 new case time series. Factors that influenced the lag were further examined by both multiple linear regression model and spatial autoregressive model. We found that (1) human mobility is positively associated with COVID-19 transmission with a median time lag of 10 days (interquartile range 8 – 11 days) and correlation coefficient of 0.68(±0.12). (2) The time lag is shorter in cities with more population flow from Wuhan, better medical resources, and denser urban road network but longer in economically advantaged cities. In practice, based on the factors influencing the time lag, the implications for policy making in controlling COVID-19 and other potential infectious diseases in the future are as follows:

(1) Cities with developed economy should maintain a high degree of sensitivity to launch the emergency response as soon as sporadic cases are detected, avoiding a rapid initial spread and community transmission.
(2) In the event of an outbreak, cities with high road network density and compact urban structure should decisively implement mobility restrictions measures, which can reduce the transmission of infectious disease in a relatively short period of time. In cities with loose structure, mobility restrictions play a limited role, therefore it is important to strengthen the implementation of other measures such as contact tracing of suspected ill persons and confirmed cases, personal preventive actions and so on.
(3) A shortage of medical resources such as detection kits and hospital capacity will inevitably prolong diagnosis time, which results in a slower response speed. So proper scheduling and allocation of medical resources is critical.

This study is subject to several limitations. First, in this study, we only considered the effect of mobility on the cases, other factors, such as the protection of personal hygiene and the hard work of the front-line fighters, were not in the model due to the lack of city-scale data. Therefore some cities decline trend in new case could not be explained well by the declining mobility. These factors are also critical for our understanding of COVID-19 transmission and need further exploration. Second, the case data might be subject to errors due to both reporting issues and limited testing capacity. These might also be different across the cities. While we partially addressed this issue by using a 3-day moving average of the case data, some uncertainty still remains. Third, due to the different request frequency in different cities, there may be selection bias in the Baidu mobility index and the users are still underrepresented among specific subgroups (i.e., children and the elderly). Fourth, we did not distinguish the trip purpose, since different destinations have different risk of infection, it is interesting to explore that which purpose of travel reduction is more effective for controlling the outbreak. In the future, we plan to collect more detail trajectory data within city to understand it.

## References

[1] Q. Li, “Early transmission dynamics in Wuhan, China, of novel coronavirus-infected pneumonia,” New England J. Med., vol. 382, pp. 1199–1207, Mar. 2020. 31995857 10.1056/NEJMoa2001316 PMC7121484

[2] N. Zhu, D. Zhang, W. Wang, X. Li, B. Yang, J. Song, X. Zhao, B. Huang, W. Shi, R. Lu, P. Niu, F. Zhan, X. Ma, D. Wang, W. Xu, G. Wu, G. F. Gao, D. Phil, W. Tan, and P. Niu, “A novel coronavirus from patients with pneumonia in China, 2019,” New Eng. J. Med., vol. 382, no. 8, pp. 727–733, Feb. 2020. 31978945 10.1056/NEJMoa2001017 PMC7092803

[3] World Health Organization. WHO Coronavirus Disease (COVID-19) Dashboard. World Health Organization. Accessed: Oct. 27, 2020. [Online]. Available: https://covid19.who.int/

[4] N. G. Davies, “Effects of non-pharmaceutical interventions on COVID-19 cases, deaths, and demand for hospital services in the UK: A modelling study,” Lancet Public Health, vol. 5, no. 7, pp. e375–e385, Jul. 2020. 32502389 10.1016/S2468-2667(20)30133-X PMC7266572

[5] S. Flaxman, I. College COVID-19 Response Team, S. Mishra, A. Gandy, H. J. T. Unwin, T. A. Mellan, H. Coupland, C. Whittaker, H. Zhu, T. Berah, J. W. Eaton, M. Monod, A. C. Ghani, C. A. Donnelly, S. Riley, M. A. C. Vollmer, N. M. Ferguson, L. C. Okell, and S. Bhatt, “Estimating the effects of non-pharmaceutical interventions on COVID-19 in Europe,” Nature, vol. 584, no. 7820, pp. 257–261, Aug. 2020. 32512579 10.1038/s41586-020-2405-7

[6] S. Lai, N. W. Ruktanonchai, L. Zhou, O. Prosper, W. Luo, J. R. Floyd, A. Wesolowski, M. Santillana, C. Zhang, X. Du, H. Yu, and A. J. Tatem, “Effect of non-pharmaceutical interventions to contain COVID-19 in China,” Nature, vol. 585, no. 7825, pp. 410–413, Sep. 2020. 32365354 10.1038/s41586-020-2293-x PMC7116778

[7] M. U. G. Kraemer, C.-H. Yang, B. Gutierrez, C.-H. Wu, B. Klein, D. M. Pigott, L. du Plessis, N. R. Faria, R. Li, W. P. Hanage, J. S. Brownstein, M. Layan, A. Vespignani, H. Tian, C. Dye, O. G. Pybus, and S. V. Scarpino, “The effect of human mobility and control measures on the COVID-19 epidemic in China,” Science, vol. 368, no. 6490, pp. 493–497, 5 2020. 32213647 10.1126/science.abb4218 PMC7146642

[8] K. Linka, M. Peirlinck, F. Sahli Costabal, and E. Kuhl, “Outbreak dynamics of COVID-19 in europe and the effect of travel restrictions,” Comput. Methods Biomech. Biomed. Eng., vol. 23, no. 11, pp. 710–717, Aug. 2020. 10.1080/10255842.2020.1759560 PMC7429245 32367739

[9] H. Tian, “An investigation of transmission control measures during the first 50 days of the COVID-19 epidemic in China,” Science, vol. 368, no. 6491, pp. 638–642, 5 2020. 32234804 10.1126/science.abb6105 PMC7164389

[10] A. Wesolowski, C. O. Buckee, K. Engø-Monsen, and C. J. E. Metcalf, “Connecting mobility to infectious diseases: The promise and limits of mobile phone data,” J. Infectious Diseases, vol. 214, no. 4, pp. S414–S420, Dec. 2016. 28830104 10.1093/infdis/jiw273 PMC5144902

[11] Q. Zhang, K. Sun, M. Chinazzi, A. P. y Piontti, N. E. Dean, D. P. Rojas, S. Merler, D. Mistry, P. Poletti, L. Rossi, M. Bray, M. E. Halloran, I. M. Longini, Jr., and A. Vespignani, “Spread of Zika virus in the Americas,” Proc. Natl. Acad. Sci. USA., vol. 114, no. 22, pp. E4334–E4343, 5 2017. 28442561 10.1073/pnas.1620161114 PMC5465916

[12] V. Colizza, A. Barrat, M. Barthélemy, and A. Vespignani, “The role of the airline transportation network in the prediction and predictability of global epidemics,” Proc. Nat. Acad. Sci. USA, vol. 103, no. 7, pp. 2015–2020, Feb. 2006. 16461461 10.1073/pnas.0510525103 PMC1413717

[13] E. Massaro, D. Kondor, and C. Ratti, “Assessing the interplay between human mobility and mosquito borne diseases in urban environments,” Sci. Rep., vol. 9, no. 1, Nov. 2019, Art. no. 16911. 10.1038/s41598-019-53127-z PMC6858332 31729435

[14] J. S. Jia, X. Lu, Y. Yuan, G. Xu, J. Jia, and N. A. Christakis, “Population flow drives spatio-temporal distribution of COVID-19 in China,” Nature, vol. 582, no. 7812, pp. 389–394, Jun. 2020. 32349120 10.1038/s41586-020-2284-y

[15] M. Chinazzi, J. T. Davis, M. Ajelli, C. Gioannini, M. Litvinova, S. Merler, A. Pastore Y Piontti, K. Mu, L. Rossi, K. Sun, C. Viboud, X. Xiong, H. Yu, M. E. Halloran, I. M. Longini, and A. Vespignani, “The effect of travel restrictions on the spread of the 2019 novel coronavirus (COVID-19) outbreak,” Science, vol. 368, no. 6489, pp. 395–400, Apr. 2020. 32144116 10.1126/science.aba9757 PMC7164386

[16] M. Gatto, E. Bertuzzo, L. Mari, S. Miccoli, L. Carraro, R. Casagrandi, and A. Rinaldo, “Spread and dynamics of the COVID-19 epidemic in italy: Effects of emergency containment measures,” Proc. Nat. Acad. Sci. USA, vol. 117, no. 19, pp. 10484–10491, 5 2020. 32327608 10.1073/pnas.2004978117 PMC7229754

[17] H. S. Badr, H. Du, M. Marshall, E. Dong, M. M. Squire, and L. M. Gardner, “Association between mobility patterns and COVID-19 transmission in the USA: A mathematical modelling study,” Lancet Infectious Diseases, vol. 20, no. 11, pp. 1247–1254, 2020, doi: 10.1016/S1473-3099(20)30553-3. 32621869 PMC7329287

[18] L. Chen, J. Cai, Q. Lin, B. Xiang, and T. Ren, “Imported COVID-19 cases pose new challenges for China,” J. Infection, vol. 80, no. 6, pp. e43–e44, Jun. 2020. 10.1016/j.jinf.2020.03.048 PMC7151481 32283157

[19] Aurora Big Data. Mobile Map App Research Report: Which of the Highest, the Baidu, and Tencent Is Strong. 36Kr. Accessed: Oct. 27, 2020. [Online]. Available: https://baijiahao.baidu.com/s?id=1590386747028939917&wfr=spider&for=pc

[20] K. Leung, J. T. Wu, D. Liu, and G. M. Leung, “First-wave COVID-19 transmissibility and severity in China outside hubei after control measures, and second-wave scenario planning: A modelling impact assessment,” Lancet, vol. 395, no. 10233, pp. 1382–1393, Apr. 2020. 32277878 10.1016/S0140-6736(20)30746-7 PMC7195331

[21] J. Zhang, M. Litvinova, Y. Liang, Y. Wang, W. Wang, S. Zhao, Q. Wu, S. Merler, C. Viboud, A. Vespignani, M. Ajelli, and H. Yu, “Changes in contact patterns shape the dynamics of the COVID-19 outbreak in China,” Science, vol. 368, no. 6498, pp. 1481–1486, Jun. 2020. 32350060 10.1126/science.abb8001 PMC7199529

[22] Z. Fan, Q. Zhan, C. Yang, H. Liu, and M. Zhan, “How did distribution patterns of particulate matter air pollution (PM_2.5_ and PM₁₀) change in China during the COVID-19 outbreak: A spatiotemporal investigation at chinese city-level,” Int. J. Environ. Res. Public Health, vol. 17, no. 17, p. 6274, Aug. 2020. 10.3390/ijerph17176274 PMC7503249 32872261

[23] X. Li, P. Gong, Y. Zhou, J. Wang, Y. Bai, B. Chen, T. Hu, Y. Xiao, B. Xu, J. Yang, and X. Liu, “Mapping global urban boundaries from the global artificial impervious area (GAIA) data,” Environ. Res. Lett., vol. 15, no. 9, Aug. 2020, Art. no. 094044.

[24] P. Gong, X. Li, J. Wang, Y. Bai, B. Chen, T. Hu, X. Liu, B. Xu, J. Yang, W. Zhang, and Y. Zhou, “Annual maps of global artificial impervious area (GAIA) between 1985 and 2018,” Remote Sens. Environ., vol. 236, Jan. 2020, Art. no. 111510.

[25] C. Libiseller and A. Grimvall, “Performance of partial mann-Kendall tests for trend detection in the presence of covariates,” Environmetrics, vol. 13, no. 1, pp. 71–84, Feb. 2002.

[26] R. H. Shumway and D. S. Stoffer, “Characteristics of Time Series,” in Time Series Analysis and its Applications: With R Examples. New York, NY, USA: Springer, 2017, pp. 1–33.

[27] M. Effenberger, A. Kronbichler, J. I. Shin, G. Mayer, H. Tilg, and P. Perco, “Association of the COVID-19 pandemic with Internet search volumes: A Google trends (TM) analysis,” Int. J. Infect. Dis., vol. 95, pp. 192–197, Jun. 2020. 32305520 10.1016/j.ijid.2020.04.033 PMC7162745

[28] T. Kang, S. Yang, J. Bu, J. Chen, and Y. Gao, “Quantitative assessment for the dynamics of the main ecosystem services and their interactions in the northwestern arid area, China,” Sustainability, vol. 12, no. 3, p. 803, Jan. 2020.

[29] N. Chen, M. Zhou, X. Dong, J. Qu, F. Gong, Y. Han, Y. Qiu, J. Wang, Y. Liu, Y. Wei, J. Xia, T. Yu, X. Zhang, and L. Zhang, “Epidemiological and clinical characteristics of 99 cases of 2019 novel coronavirus pneumonia in wuhan, China: A descriptive study,” Lancet, vol. 395, no. 10223, pp. 507–513, Feb. 2020. 32007143 10.1016/S0140-6736(20)30211-7 PMC7135076

[30] J. F. W. Chan, “A familial cluster of pneumonia associated with the 2019 novel coronavirus indicating person-to-person transmission: A study of a family cluster,” Lancet, vol. 395, no. 10223, pp. 514–523, Feb. 2020. 31986261 10.1016/S0140-6736(20)30154-9 PMC7159286

[31] WHO. Report of the WHO-China Joint Mission on Coronavirus Disease 2019 (COVID-19). Feb. 24, 2020. [Online]. Available: https://www.who.int/docs/default-source/coronaviruse/who-china-joint-mission-on-covid-19-final-report.pdf

[32] J. Wang, Y. Deng, C. Song, and D. Tian, “Measuring time accessibility and its spatial characteristics in the urban areas of beijing,” J. Geograph. Sci., vol. 26, no. 12, pp. 1754–1768, Nov. 2016.

[33] A. Bassolas, H. Barbosa-Filho, B. Dickinson, X. Dotiwalla, P. Eastham, R. Gallotti, G. Ghoshal, B. Gipson, S. A. Hazarie, H. Kautz, O. Kucuktunc, A. Lieber, A. Sadilek, and J. J. Ramasco, “Hierarchical organization of urban mobility and its connection with city livability,” Nature Commun., vol. 10, no. 1, p. 4817, Oct. 2019. 31645563 10.1038/s41467-019-12809-y PMC6811587

[34] L. Anselin, “Spatial externalities, spatial multipliers, and spatial econometrics,” Int. Regional Sci. Rev., vol. 26, no. 2, pp. 153–166, Apr. 2003.

[35] L. Anselin, Spatial Econometrics: Methods and Models. Dordrecht, The Netherlands: Kluwer, 1988.

[36] J. Zhang, “Evolving epidemiology and transmission dynamics of coronavirus disease 2019 outside hubei province, China: A descriptive and modelling study,” Lancet Infectious Diseases, vol. 20, no. 7, pp. 793–802, Jul. 2020. 32247326 10.1016/S1473-3099(20)30230-9 PMC7269887

[37] Y. Qiu, X. Chen, and W. Shi, “Impacts of social and economic factors on the transmission of coronavirus disease 2019 (COVID-19) in China,” J. Popul. Econ., vol. 33, no. 4, pp. 1127–1172, Oct. 2020. 10.1007/s00148-020-00778-2 PMC7210464 32395017

[38] S. M. Moghadas, A. Shoukat, M. C. Fitzpatrick, C. R. Wells, P. Sah, A. Pandey, J. D. Sachs, Z. Wang, L. A. Meyers, B. H. Singer, and A. P. Galvani, “Projecting hospital utilization during the COVID-19 outbreaks in the united states,” Proc. Nat. Acad. Sci. USA, vol. 117, no. 16, pp. 9122–9126, Apr. 2020. 32245814 10.1073/pnas.2004064117 PMC7183199

[39] J. Aguilar, A. Bassolas, G. Ghoshal, S. Hazarie, A. Kirkley, M. Mazzoli, S. Meloni, S. Mimar, V. Nicosia, J. J. Ramasco, and A. Sadilek, “Impact of urban structure on COVID-19 spread,” 2020, arXiv:2007.15367. [Online]. Available: http://arxiv.org/abs/2007.15367 10.1038/s41598-022-06720-8 PMC8907266 35264587

[40] F. Gong, Y. Xiong, J. Xiao, L. Lin, X. Liu, D. Wang, and X. Li, “China’s local governments are combating COVID-19 with unprecedented responses—From a wenzhou governance perspective,” Frontiers Med., vol. 14, no. 2, pp. 220–224, Apr. 2020. 10.1007/s11684-020-0755-z PMC7089477 32166600

[41] X. Yu and R. Yang, “COVID-19 transmission through asymptomatic carriers is a challenge to containment,” Influenza Other Respiratory Viruses, vol. 14, no. 4, pp. 474–475, Jul. 2020. 32246886 10.1111/irv.12743 PMC7228388

